# Women carry for less: body size, pelvis width, loading position and energetics

**DOI:** 10.1017/ehs.2022.35

**Published:** 2022-08-04

**Authors:** Cara M. Wall-Scheffler

**Affiliations:** Department of Biology, Seattle Pacific University, Seattle, WA, USA and Department of Anthropology, University of Washington, Seattle, WA, USA

**Keywords:** Cost of transport, cost of locomotion, center of mass, sexual dimorphism, obstetrical dilemma

## Abstract

The energetic cost of walking varies with mass and speed; however, the metabolic cost of carrying loads has not consistently increased proportionally to the mass carried. The cost of carrying mass, and the speed at which human walkers carry this mass, has been shown to vary with load position and load description (e.g. child vs. groceries). Additionally, the preponderance of women carriers around the world, and the tendency for certain kinds of population-level sexual dimorphism has led to the hypothesis that women might be more effective carriers than men. Here, I investigate the energetic cost and speed changes of women (*N =* 9) and men (*N =* 6) walking through the woods carrying their own babies (mean baby mass = 10.6 kg) in three different positions – on their front, side and back using the same Ergo fabric baby sling. People carrying their babies on their backs are able to maintain their unloaded walking speed (1.4 m/s) and show the lowest increase in metabolic cost per distance (J/m, 17.4%). Women carry the babies for a lower energetic cost than men at all conditions (*p* < 0.01). Further energetic and kinematic evidence elucidates the preponderance of back-carrying cross-culturally, and illustrates the importance of relatively wider bi-trochanteric breadths for reducing the energetic costs of carrying.

## Graphical abstract

**Figure:**
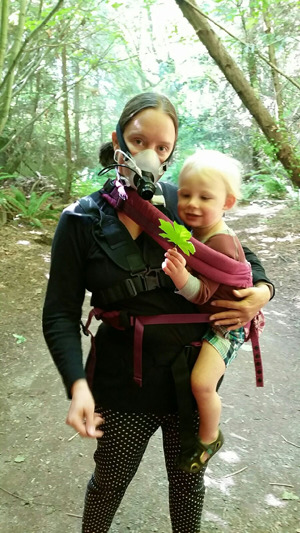

**Social media summary:** This is the first study to investigate people carrying babies across challenging terrain; women have clear advantages.

## Introduction

There are few universals among living populations of humans, but load carrying, and particularly baby-carrying, is one of these universals (Rosenberg et al., 2004; Wall-Scheffler et al., 2007); however, the manner in which children are carried shows some variation. This variation can be related to textiles and materials used to ‘sling’ children (van Hout, 2011), but it can also be present in the location on the body where children are carried (Wall-Scheffler & Myers, 2013, 2017). There is reason to believe that slinging location – and in fact the position of any burden – may change the metabolic cost of carrying an item of a given mass in parallel with changes to the mechanical work to be done (Myers & Steudel, 1985).

**Figure 1.**
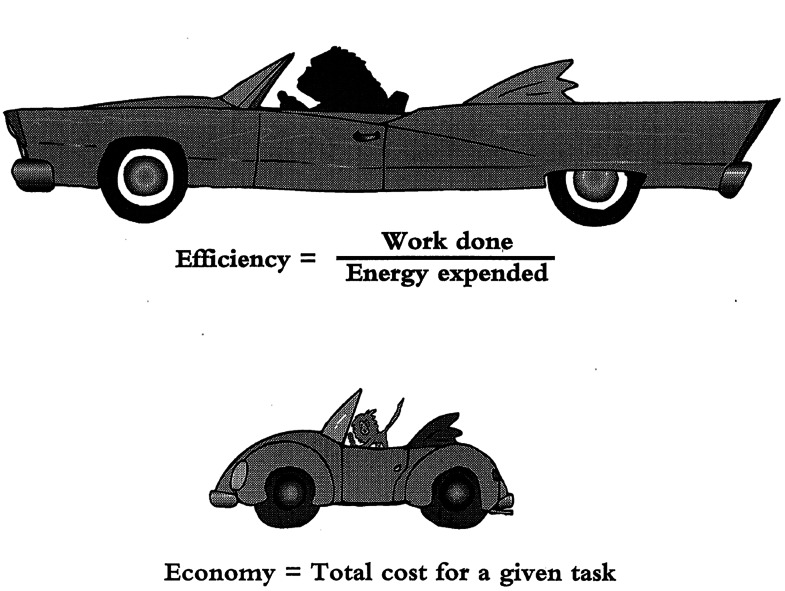
In animal physiology, efficiency and economy are considered evolutionary trade-offs. If an organism is large (for a variety of reasons, such as Bergmann's Rule or predator avoidance), they have ‘chosen’ the metabolic strategy of efficiency. Their total cost is high, but their cost per kg is low, making their locomotor activities ‘efficient’. If an organism is small (different strategy for the same pressures above, and also because reliance on making somatic tissue can distract from reproduction), then their total cost is low, making them energetically economical, even though their cost per kilogram is quite high. In situations of sexual ‘dimorphism’, as is typical of human populations on average, females are considered economical, but not efficient. Recent work that has carefully tested both economy and efficiency actually shows that not only are women dramatically more economical than men under all locomotor conditions, but they are also equally, or even more, efficient, particularly when carrying loads and walking on inclines.

Additionally, in the last few years, numerous paradigms have been challenged, and what we think about how labour is distributed and who does specific tasks has been shifting (Bennett et al., 2020; Fuentes, 2021; Khorasani & Lee, 2020). Papers pushing away from sexual ‘di'morphism (Sitek et al., 2012), a gendered division of labour (Bennett et al., 2020; Khorasani & Lee, 2020), and reproductive constraints on morphology (Dunsworth, 2021; Dunsworth et al., 2012; Wall-Scheffler & Myers, 2017), have all been investigating what we actually know about human biological and cultural variation, and what we have assumed to be true based on nineteenth and twentieth century cultural norms in Europe and North America. For example, colleagues and I have argued that women are excellent (i.e. both economical and efficient) load carriers (and walkers generally; Wall-Scheffler et al., 2007; Wall-Scheffler & Myers, 2013, 2017), whereas others have argued that there are not sexually dimorphic characters that influence locomotor energetics or kinematics in one way or another (Prado-Novoa et al., 2019; Warrener et al., 2015). Part of this latter issue really has to do with how the energetic cost of locomotion is calculated, and some differences in the way in which people compare and contrast efficiency (the energy required to do work, variously defined as moving mass a given distance) and energy economy (the total cost to complete a task; Steudel, 1994; Figure 1). Conflating efficiency and economy, and assuming some semblance of geometric similarity in humans of different sizes (which does not in fact exist; Kramer & Sylvester, 2009, 2013) have created some recent difficulties in understanding the energetic impact of load carrying, and whether in fact load carrying has acted as a selection pressure by influencing the metabolic burden enough that differential fertility results (Gibson & Mace, 2006).

Metabolic efficiency in particular can be difficult to define, and here I balance the functional definitions from Steudel (1994) with those of Kramer and Sylvester (2009). There are numerous ways to measure metabolic efficiency using mechanical work, although these work estimates can differ substantially depending upon what assumptions are made regarding the extent of energy exchange between limb segments (Zelik & Kuo, 2010). Steudel (1994) argues that both cost of locomotion (COL, cost per unit time) and cost of transport (COT, cost per unit distance) can be measures of ‘locomotor metabolic efficiency’ when we consider these as work done for a given mass (Figure 1). Kramer and Sylvester (2009) note in their Table 1 that efficiency is indeed a measure of the metabolic energy used per unit mass (‘for a given task’), but focus primarily on COT, and not COL (which they focus on as an absolute measure of economy). Here, all values will be used – COT, COL and mass-specific COT – in order to explicate how energy for a given task can be compared, and most importantly, understood from an evolutionary perspective. Although the word economy might sometimes be used colloquially instead of efficiency, thus leading to confusion in interpretation, it is clear that when used correctly, economy should specifically refer to the total energy used for a given task, and is not a term to be used for data that are mass specific (or for data on mechanical work; Kramer & Sylvester, 2009; Steudel, 1994). In ecological and field-based situations, efficiency is often measured over the entire task, using mass and distance travelled as the measures of work, and efficiency is thus reported as mass-specific COT, for example (e.g. Kramer & Sylvester, 2009). This might be in contrast to measures taken in a laboratory, where mechanical work over a gait cycle is measured by changes in force production, and efficiency is thus reported as mechanical work divided by metabolic cost (e.g. Cavagna & Margaria, 1966).

**Table 1.** The means and standard deviations of cost-related variables of loaded walking

	W		J/m		J/m/kg (body mass)		J/m/kg (total work)	
	Mean	Standard deviation	Mean	Standard deviation	Mean	Standard deviation	Mean	Standard deviation
Unloaded	453.5	153.0	310.1	85.3	4.1	0.7	3.6	0.6
Front	542.9	165.1	375.6	85.2	5.0	0.6	4.3	0.5
Side	533.3	183.1	372.9	104.2	4.9	0.8	4.3	0.7
Back	535.6	171.3	364.0	93.0	4.7	0.6	4.1	0.5

From an evolutionary perspective, both efficiency and economy are important variables that have the potential to influence size and shape, not to mention mobility strategies, by shifting reproductive success. For example, economical benefits of small(er) body size mean protection against food shortages (Diamond, 1991) and less energy spent on somatic growth that can instead be spent on reproduction, particularly in challenging niches (Migliano et al., 2007). Increased energetic economy further increases endurance and the ability to travel longer distances (Conley & Krahenbuhl, 1980). In addition, the small body size associated with increased economy is also accompanied generally by a higher surface area to volume ratio, meaning a smaller heat gain, and a more rapid dispersal of that heat (Wall-Scheffler, 2015) which can subsequently further increase endurance. To this end, economy influences numerous variables of importance to selection, particularly in our lineage.

Efficiency similarly has crucial mobility benefits. A larger body size among hominins means that longer strides can be taken, which means that distances can be covered in fewer steps (Steudel-Numbers, 2006; Steudel-Numbers & Tilkens, 2004; Steudel, 1994, 1996). Given that the cost of walking can be formulated as the cost of taking a step, and that this is then extrapolated across the distance travelled (Donelan et al., 2002; Selinger et al., 2015; Soo & Donelan, 2010), minimising the cost of taking a step for a given mass can dramatically decrease the overall cost of locomotion, relative to body size, while still allowing for increased distances travelled (Foley, 1987). Larger body sizes also increase the loads that can be carried, subsequently increasing the age at which children are carried by (allo-)parents (Aiello & Wells, 2002; Bouterse & Wall-Scheffler, 2018; Kramer, 1998). Larger body size alone can cause some problems for bipeds (Aiello & Wells, 2002), particularly in increasing heat loads, so additional changes, like longer lower limbs, will be important to maintaining locomotor-specific efficiencies (Steudel-Numbers & Tilkens, 2004; Wall-Scheffler, 2014).

To this end, I seek to provide some answers to the problems raised above, as well as a series of complex questions in the study of human mobility, reproduction and energetics by investigating the metabolic costs and kinematic outcomes of walking humans carrying their own babies overground in a forest. Carrying overground lends numerous important variables, including changes in terrain like inclines, which will be specifically investigated here. Additionally, because people around the world carry their babies and other loads in different positions (Wall-Scheffler & Myers, 2013) and at different developmental stages (Bouterse & Wall-Scheffler, 2018), I further investigate how loading position changes key outcome variables, particularly speed and these different measures of metabolic energy. Given past research, I expect to find that people carry loads with an increased cost proportional to the percentage of the load to their total body mass, while also showing that women carry loads for a lower energetic cost than men (Wall-Scheffler, 2012a, b; Wall-Scheffler & Myers, 2013, 2017). I also expect that traversing variable terrain will increase the metabolic cost above metabolic expenditures for level treadmill walking (Passmore & Durnin, 1955; Voloshina et al., 2013).

## Methods

Nine women and six men were asked to walk along a forest path under a series of different loading conditions: unloaded and then carrying their own baby on their side, front and back in a randomised order. Side carrying involves the baby being placed essentially on the hip, with one leg around the back of the parent's body and one leg around the front. The parent's arm extends around the baby's body bu does not support the baby's mass (because of the sling). Each of these positions used the same sling (an ergobaby^©^ soft baby carrier, configured slightly differently for the different positions). The sling weighed 640 g. For the purpose of this study, all parents were the biological parents of their babies, and the mothers had breastfed the babies. Parent mass, baby mass, stature, lower limb length, bi-iliac breadth and bi-trochanteric breadth were all measured immediately prior to the study following already published methods (Wall-Scheffler et al., 2006). All parents reported having engaged in regular child carrying during their baby's life prior to the study, although not always with a fabric sling. Most parents had used a sling or some carrying device for back and front carrying, but had not used a sling specifically for side carrying. Side carrying was typically accomplished by bracing the baby against the side with an arm. Babies alternated between being asleep and awake at various portions of the trial (which could take 90 min). Typically the babies were asleep while the parent was walking and awake during the breaks between walking conditions.

There were no significant differences between mothers and fathers in terms of their ages (mothers, 36.4 ± 4.0 years; fathers, 37.0 ± 3.6 years), the ages of the babies they carried (mothers, 16.4 ± 7.4 months; fathers, 14.8 ± 6.4 months) or the mass of the babies (mothers, 11.0 ± 3.1 kg; fathers, 10.2 ± 2.4 kg). There were significant differences in some measures of body size between mothers and fathers, however. The fathers had significantly higher masses (mothers, 66.9 ± 5.3 kg; fathers, 86.0 ± 18.4 kg; *p* = 0.01) and were taller (mothers, 168.5 ± 2.7 cm; fathers, 181.0 ± 7.8 cm; *p* = 0.01) than the mothers as is typical on average across human populations. Despite this, a;though the mothers carried babies of a slightly larger percentage of their mass than fathers (16.4% vs. 12.3%), this difference was not significant (*p* = 0.103), even when taking into account the additional mass of the sling itself (*p* = 0.093). In this sample, fathers did have significantly wider pelves in absolute measures for both bi-iliac (*p* = 0.01) and bi-trochanteric breadth (*p* = 0.045) (since again, they were relatively ‘bigger’), but these differences went away relative to stature (*p* > 0.350). Relative to mass, women had the wider pelves (both bi-iliac and bi-trochanteric) (*p* < 0.05). Thus, in this sample we had both size and shape (e.g. relative proportions) differences between women and men that are typical of many human populations. In these comparisons, ‘relative to’ was defined as the percentage of the size variable (e.g. mass, stature). All participants signed written informed consent forms approved by the Institutional Review Board of Seattle Pacific University to participate in the study.

### Energetic data

**Figure 2.**
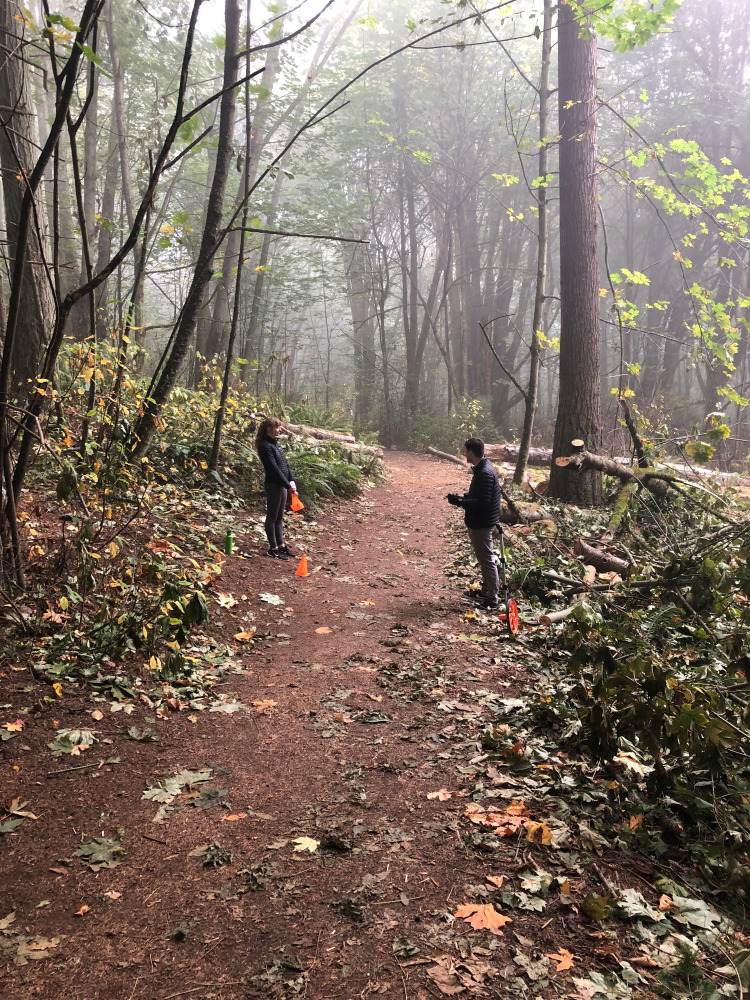
Data collection taking place along the forest path.

All participants walked roughly 1 km along a temperate forest path (Figure 2) while unloaded, and at each of the loaded conditions in a randomised order. The path varies in width (from 0.5 to 1.5 m) and winds through the forest with inclines, declines and curves, but no switchbacks; the average grade is about 1.0% and is thus fairly balanced with incline and decline. Along the path edges ground cover includes ferns, salal, nettles and native rose. Trees can be up to the path edge, and roots can cross the paths, making the ground somewhat uneven in patches. Tree species include big-leaf maples (*Acer macrophyllum*), western red cedar (*Thuja plicata*) and Douglas fir (*Pseudotsuga menzeseii*). Leaves, branches and needles from each of these species also cover the walking path.

Each walking condition was along the same path, so participants repeated the protocol with each loading position, with at least 10 min between each condition. Participants rested in a chair for 10 min prior to the start of the study, so in order to begin the next speed, their metabolic rate needed to be back to their initial resting value. A participant's metabolic rate was measured in a breath-by-breath fashion using a Cosmed K4b2 Metabolic Mobile Testing System for the entirety of the 1 km path, although the average of the last 2 min of walking was used here to ensure only steady-state data were included (Steudel-Numbers & Tilkens, 2004; Steudel-Numbers et al., 2007). Speed was calculated by dividing the exact distance each participant walked by the total time they took to walk the path. Distance was measured using a Keson measuring wheel. The COL was recorded from the final 2 min of each condition in Watts. Additionally, COT was also calculated as Joules per metre (i.e. COL divided by speed). In order to better understand the tension between size and shape, the mass-specific COT was also calculated, in terms of both Joules per metre per body mass of the carrier and Joules per metre per total mass being moved (carrier + baby).

At all times, the participant wore the Cosmed K4b2, which weighed 1.24 kg. The ergobaby© sling was only worn when the baby was being carried, thus adding 0.64 kg to the baby's mass, but not to the unloaded condition.

### Kinematic data

For a small subportion of the path, which included a level area and an inclined area (7.6% incline; each about 10 m long), kinematics were also recorded, as was speed at these specific zones. Video of at least six strides per condition was used to calculate contact and swing times; the average of the six strides was used to calculate total stride time for each condition. After this, contact and swing times as a percentage of total stride time were calculated. Owing to equipment malfunction in the field, video is not available for all participants. The sample size for contact and swing times is four women and five men. Stop watches were also used to measure stride frequency and then calculate stride length for all participants.

### Statistics

All statistics were done in SPSS 28.0, using repeated measures ANOVAs for each of the dependent, metabolic variables, with gender as a fixed factor. ‘Load position’ was the within-subjects factor of the repeated measure. For the kinematic analysis investigating load and incline, ‘incline’ was an additional within-subjects factor.

Additionally, to quantify the importance of size and shape, linear regressions predicting metabolic cost were also run, with anthropometrics (stature, lower limb length, bi-iliac breadth and bi-trochanteric breadth) as predictive covariates. In order to parse out what might be driving cost at the three different loading positions, the metabolic rate at each loaded position was normalised to the unloaded walking by calculating the percentage change of the loaded metabolic rate from the unloaded walking. Linear regressions were then run for each normalised cost variable (COL or COT) and each model included the load position and the mass of the infant in addition to anthropometrics.

## Results

### Energetics

**Figure 3.**
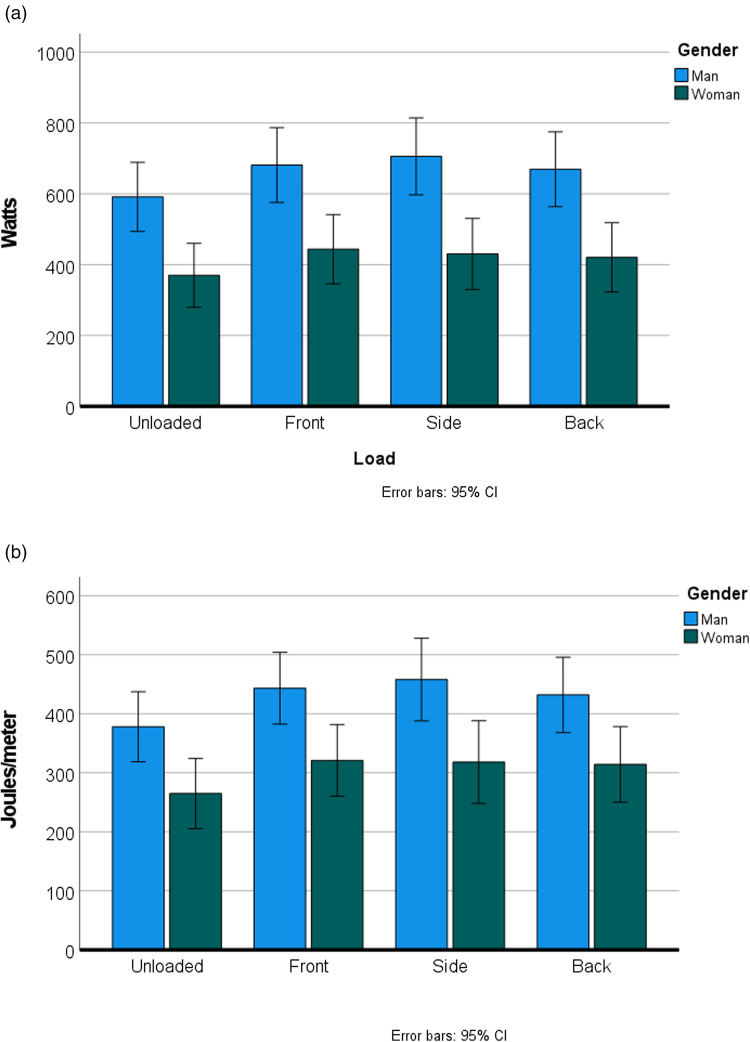
(a) The cost of locomotion (W) at each of the loading positions. (b) The cost of transport (J/m) at each of the loading positions. Women are significantly more (*p* = 0.01) economical than men, both in cost per unit time (W), as well as in cost per unit distance (J/m).

**Figure 4.**
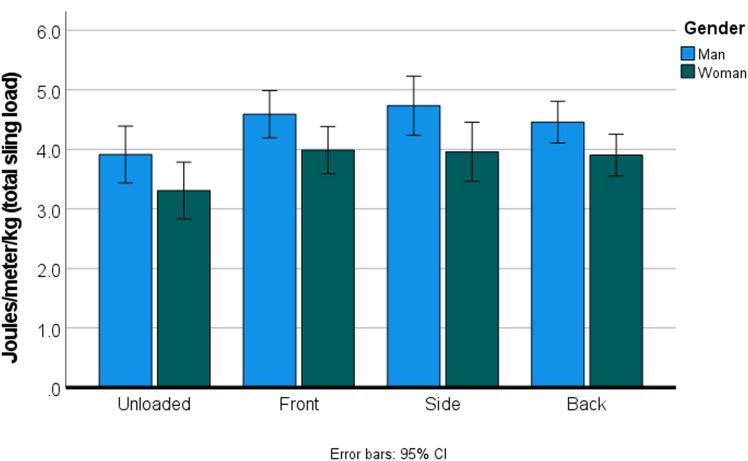
Women were significantly more efficient than men at all walking conditions (*p* = 0.035).

The baby plus sling mass was on average 15.7% of the mass of the walkers. Carrying this load did not significantly change the overall speed of the route (*p* = 0.164), although the speed did decrease slightly for front and side loading (2.2%, from 1.44 to 1.41 m/s). For the entire sample, carrying the load significantly increased energetic cost (both COL and COT), regardless of the loading position (*p* < 0.001) and regardless of whether the COT was mass specific or not (Table 1; Figure 3). There were no significant differences between the different loading positions and metabolic cost, but back loading showed the lowest increase in COT, with an absolute increase of 17.4%, vs. 21.1% for front loading (Figure 3b). For the mass-specific COT, back loading only increased the COT by 15.1%, in comparison with 20.8% for front loading. For the total load carried, back loading increased the COT by 14.8%; front loading still increased the COT by 20.3% (Figure 4). Side loading is similar to front loading, which oscillates around a 20% cost increase.

At all conditions women were significantly more economical (i.e. used significantly less energy than men to complete the task), both in terms of COL (W, *p* = 0.002, Figure 3a), and in terms of COT (J/m, *p* = 0.011, Figure 3b). Women were also significantly more efficient (i.e. used significantly less energy per kg of mass moved) than men (J/m/total kg, *p* = 0.035, Figure 4).

### Anthropometrics

A regression model was run to determine the relationship between different body proportions on metabolic cost across the different loaded positions. Three variables were used to help understand load position: the position itself, the mass of the infant and the interaction between the infant's mass and the position. Four variables were used to help understand the effect of body shape on metabolic cost: stature, lower limb length, bi-iliac breadth and bi-trochanteric breadth. COL and COT at the loaded positions were normalised to unloaded walking in order to more clearly parse out what anthropometrics might help modulate carrying a load at different positions on the body. Since speed did not significantly vary between the loads, both speed and body mass were already controlled by normalising to unloaded walking; as such these variables were not included in the regression models. Each regression was run in a step-wise fashion, with each variable being entered sequentially; this allows us to assess the strength of the effect of each variable by identifying the increase in the *R*^2^ value at each step.

For the model for COL (cost per unit time), load position and its interaction with the baby's mass were highly influential aspects of normalised cost. Additionally, both pelvic measures stayed in the model as highly significant (Table 2), while stature and lower limb length did not show significant affects on COL and were removed from the model. Bi-trochanteric breadth showed a negative *β*-value, indicating that as relative (to body size) pelvis breadth increases, metabolic cost decreases – this effect was extremely strong, explaining over 20% of the variation in COL. The final *R*^2^ of the model was 84.3%.

**Table 2.** The best fit linear regression results of normalised cost of locomotion (W)

	Standardised coefficients	Significance	Cumulative *R*^2^
	*β*		
(Constant)		0.142	
Load position	0.491	0.015	
Baby mass (kg)	1.248	<0.001	
Position–baby mass interaction	−0.904	0.005	53.0%
Bi-iliac (cm)	0.378	<0.001	64.1%
Bi-trochanteric (cm)	−0.452	<0.001	84.3%

**Figure 5.**
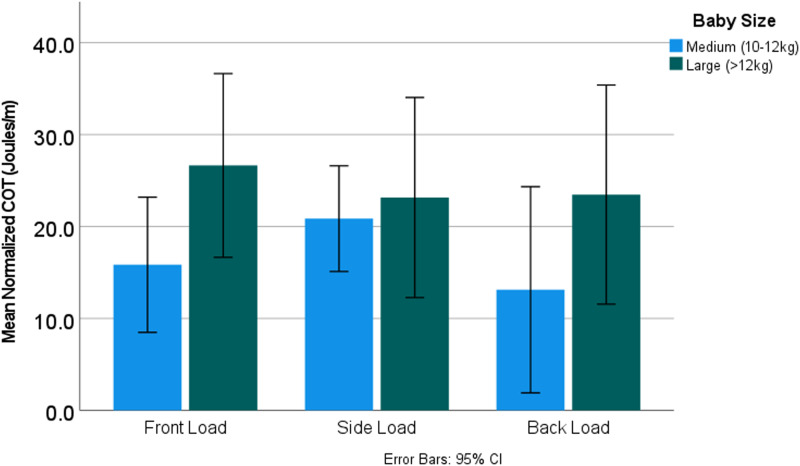
The impact of baby size on load position. The size of the baby significantly changes the relationship between loading position and normalised cost of transport (*p* = 0.036).

The COT showed a nearly identical model to the COL (Table 3). The bi-trochanteric breadth's *β*-value was again a strong, statistically significant negative value. Stature and lower-limb length did not contribute significantly to the model, whereas the infant's position in combination with its mass continued to be crucial predictors of normalised cost (Table 3; Figure 5).

**Table 3.** The best-fit regression model predicting normalised cost of transport (J/m)

	Standardised coefficients	Significance	Cumulative *R*^2^
	*β*		
(Constant)		0.901	
Load position	0.368	0.075	
Baby mass (kg)	1.185	<0.001	
Position–baby mass interaction	−0.692	0.036	65.2%
Bi-iliac (cm)	0.361	<0.001	75.4%
Bi-trochanteric (cm)	−0.299	<0.001	84.3%

The interaction between infant size and loading position (Figure 5) seems to be particularly driven by the side load position, in which even medium-sized infants incur a high increase in COT. This is in contrast to front loading, in which medium infants are effectively carried, but there is a dramatic increase in COT when heavier infants are carried on the front. Unsurprisingly, back-loaded infants incur the smallest COT increase, regardless of size.

### Kinematics

**Figure 6.**
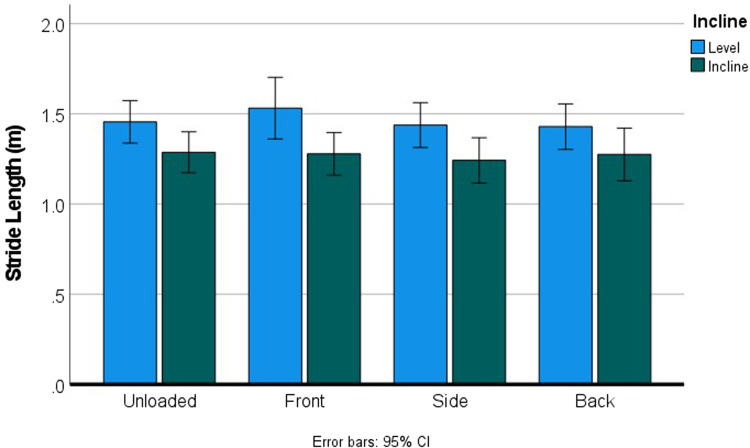
Walking on an incline significantly shortens stride length (*p* < 0.001), but carrying a load does not significantly change stride length (*p* = 0.459) under these walking conditions, nor is there a significant interaction (*p* = 0.233).

Because of the variable terrain through the forest, we are able to look at the interactive effects between the loads and incline (7.6%) on kinematics during walking. Using a repeated measures ANOVA with incline and load as within-subject factors, incline significantly shortened stride length (*p* < 0.001) and reduced speed (*p* < 0.001), whereas load alone did not significantly change stride length or speed at any of the loaded conditions (Figure 6).

**Figure 7.**
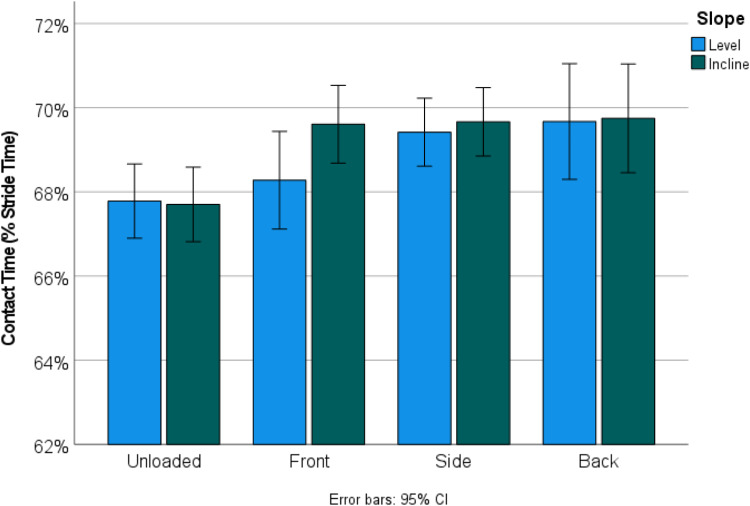
Walking on an incline did not significantly change the percentage of stride time spent in contact with the ground but being loaded significantly increased relative contact time (*p* < 0.001).

**Figure 8.**
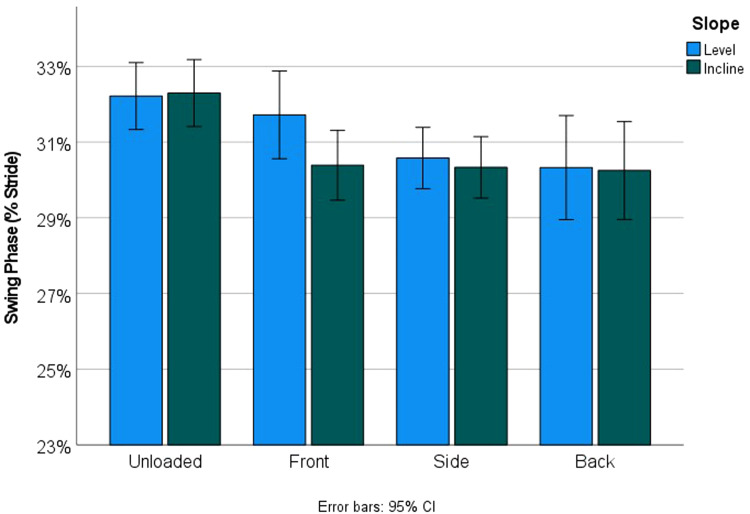
Load significantly shorted relative swing time (*p* = 0.001), though there was no significant effect of incline (*p* = 0.238).

Incline did not significantly influence relative contact time, while load significantly increased relative contact time (*p* = 0.029; Figure 7). Loading significantly shorted relative swing time (*p* = 0.001), whereas incline did not significantly influence relative swing time (*p* = 0.238; Figure 8).

This means that, although incline influenced speed and stride length (Figure 6), it did not influence the relative proportion of the stride dedicated to swing and stance phases (Table 4; Figures 7 and 8). Conversely, load did not influence speed or stride length, but did significantly perturb the relationship between swing and stance phases (Table 4; Figures 7 and 8). Kinematic patterns were similar for women and men.

**Table 4.** Kinematic changes based on the interactive effects of incline and load

	Incline	Load
Speed	Decrease	NS
Stride length (m)	Decrease	NS
Contact time (% stride)	NS	Increase
Swing time (% stride)	NS	Decrease

NS stands for Not Significant.

## Discussion

This represents one of the first papers to look at how people carry their own babies over terrain, and to specifically test how different loading positions might impact people's speed and kinematic choices. Although overall speed choices did not significantly vary across the 1000 m path, there were measurable cost differences for the different loading positions. Essentially front and side loading act similarly in increasing cost the most while simultaneously very slightly decreasing speed. These positions essentially increased cost by 20% (for a load that is 16% of total body mass) while decreasing overall speed by 2% (Wall-Scheffler & Myers, 2013). Since the terrain was variable, with substantial changes in overall incline and decline, as well as the presence of small obtrusions such as tree roots, the metabolic costs increased above that of the load itself (Rue & Kramer, 2017).

Side and front loading were further interesting because they showed dramatic, significant interactions with the size of the infant being carried. It might almost seem that side loading is the most challenging load for people in the sense that the increase in cost for carrying a load on the side existed regardless of the size of the infant – even medium-sized infants (10–12 kg) incurred a 20% increase in cost, whereas these same sized babies incurred substantially reduced costs on the front (~15%) and back (~12%) (Figure 5). However, front loading a large infant (12–14 kg) dramatically increased the cost of carrying – upwards of 25%, whereas for side loading, once you paid that 20% cost, it was essentially the same regardless of the size of the baby (Figure 5). Such a shift is clearly noticeable ethnographically, as people tend to move babies from their (the carrier's) front to their back between 9 and 15 months, which is exactly when babies, on average, move above 12 kg (cdc.gov).

In terms of what might be happening during front and side loading kinematically, the patterns of how people managed the intersection of incline and load seemed to vary (Table 4; Figures 7 and 8). There are multiple factors which could be at play here, but two seem particularly likely, and that is the way in which the body manages high-intensity activities (which might be stable) with unstable activities (of varying intensities). Incline walking is a high-intensity activity in which the net movement of the COM is lifted against gravity at every step. High-intensity activities have already been shown to decrease speed (Wall-Scheffler, 2015; Wall-Scheffler & Myers, 2013), which is exactly what we see here. In this case we see the decrease in speed coming from reducing stride length which again is typical of what other studies have found for incline walking (Higgins & Ruff, 2011). Load carrying conversely can lead to decreased stability from having mass unevenly distributed around the body (Maloiy et al., 1986; Minetti et al., 2006); the typical kinematic pattern of reduced stability is to maintain a longer contact time, as well as a wider gait (Donelan et al., 2004; Kuo, 1999). Here we clearly see a longer contact time as a means of maintaining stability during load carrying (Table 4). Perturbations between the different load positions themselves (i.e. between front and side loading) could be an indicator of the importance of infant size (as above, infant size perturbs stability during front and side loading differently), in addition to increased modulation of the central pattern generator (CPG) driving the walking cycle. It remains possible that CPGs are not as fine-tuned for front and side loads as back loads (Bertram, 2005). Without a specific modulatory neural regime, it is possible that the metabolic costs increased more with the challenge of the terrain, added with the challenge of less familiar loading patterns. Decreased modulation of the CPG could exacerbate issues of maintaining a constant pelvic tilt and torso stability during front and side loading (Faraji et al., 2018), which might explain the alterations between stance and swing phases on the level and inclined surfaces. Front loading in particular shows dramatic interactions between level and inclined walking: the swing phase dominated on a level surface, whereas the stance phase dominated while front loaded on the incline – suggesting that for front loading, walking uphill was a high intensity activity that was also unstable.

Conversely to what was happening with front and side loading, during back loading people walked (on average) at identical speeds as unloaded, and simultaneously incurred an increased cost of around 15%, directly proportional to the increased mass of the load (Maloiy et al., 1986; Taylor et al., 1980). Kinematically, during back loading people are spending relatively more time in the stance phase, and swinging the lower limb through more quickly (proportionally less time in swing phase; Figure 7 vs. Figure 8). Given that both speed and stride length stay constant, it is through these gait phase proportions that back loading seems to maintain walking effectiveness. Colleagues and I have argued previously that being able to utilise pelvic rotation to limit the distance through which the limb swings can be a way to maximise energy savings (Rak, 1991; Wall-Scheffler & Myers, 2017), potentially explaining some of the sex differences in cost (e.g. a wider pelvis allows for further limb swing, see below), in addition to why back loading might be effective. It is possible that back loading allows for the lower limb to swing through more quickly, given that there is no obstruction of the baby's body in the direction of motion, as is possible during side and front loading.

As such, this study continues to unpack the numerous advantages that specific body proportions might have on energy utilisation (Steudel-Numbers & Tilkens, 2004; Wall-Scheffler, 2014), and to further emphasise that patterns of sex-related size differences can be understood as increasing human female locomotor economy and efficiency. Here the data clearly show that mothers have energetic advantages over fathers, being both more economical (using less energy overall for the particular task) and more efficient (using relatively less energy for the amount of work being done). Given that men are more likely to stay extremely close to their optimal walking speed (Wall-Scheffler, 2012b, 2015), we expect that the men in particular were walking in such a way as to optimise their energy expenditure, thus making these differences even more striking.

In this sample women had relatively broader bi-trochanteric breadths for their mass than men, and that specific morphology was the only body proportion that significantly showed a decrease in metabolic cost (both COT and COL). Unloaded studies have found that longer lower limbs also decrease absolute COT (Steudel-Numbers & Tilkens, 2004). As has been shown previously (Wall-Scheffler, 2012a, 2014), relatively wider trochanteric breadths and relatively longer lower limbs can have additive effects, although in this study, wider bi-trochanteric breadths clearly made the largest cost-saving difference, explaining 20% of the cost variation in COL and 10% in COT. Although in this study the data did not specifically show how this different proportionality might have acted through kinematics to shape cost savings for the mothers, previous studies which controlled for speed showed that women have longer stride lengths while carrying than men (Wall-Scheffler & Myers, 2017; Whitcome et al., 2017). Having longer stride lengths can reduce the cost to go a given distance (i.e. COT) since fewer steps are taken (Biewener, 2003; Donelan et al., 2002).

Having a lower center of mass (COM) is another outcome of a relatively wider pelvis, and women have been shown to have a larger percentage of their muscle mass in their lower limbs (than their upper limbs; Wall-Scheffler & Myers, 2017), thus lowering their COM (first principles) and stabilising their loaded gait to a greater extent than men (Donelan et al., 2004; Kuo, 1999; Woo, 2015). A lower COM increases stability because the lower COM requires a higher change in direction to move a body outside of its base of support; in other words, it is more challenging to topple over when pushed or when standing on only a single limb if you have a lower COM. Increased stability during the gait cycle has been shown to directly reduce the energy expenditure of walking (Dean et al., 2007; Donelan et al., 2004; Ijmker et al., 2013). Additionally, in this study, each of these women had been recently pregnant with the baby they carried here, meaning that their lower limbs were even stronger than typical (Wall-Scheffler & Myers, 2017). Increasing their stability while also having effective transitions between stance and swing (Kuo et al., 2005; Wall-Scheffler, 2012a; Wall-Scheffler & Myers, 2017) can help explain the metabolic advantages during load carrying shown here. Specifically, it has been previously shown that people with relatively broader bi-trochanteric breadths (often women) increase their muscle activity just prior to footfall (‘collision’) and consequently have reduced muscular activity following the collision (Wall-Scheffler & Myers, 2017). Thus, by changing the timing of muscle use, reduced energy is needed to recover from the collision with the ground (Wall-Scheffler & Myers, 2017), meaning that the transitions between stance and swing can utilise less energy. An interesting area of future research for women especially would be to investigate whether the increased non-lean muscle mass that women have in their lower limbs allows for further dampening the work of the collision, and reducing the cost of the rebound even more (Zelik & Kuo, 2010). Given that soft tissue generally could be saving 14% of the work per stride (Zelik & Kuo, 2010), increased soft tissue has strong potential for explaining the combination of efficiency and economy of women found here. The importance of limb composition has been touted as one of the reasons women of the Luo and Kikuyu populations walk with loads at such a low metabolic cost (Maloiy et al., 1986) and is thus worth further investigation.

This study thus fits in closely with other work specifically showing that women, and people with relatively wider pelves, have more efficient loaded locomotion than those with more narrow pelves (Wall-Scheffler, 2012a, b, 2014, 2015; Wall-Scheffler et al., 2007; Wall-Scheffler & Myers, 2017). Studies which purport no significant differences between the energetic efficiency of women and men do not look at load carrying (e.g. Warrener et al., 2015) or look specifically at treadmill walking (e.g. Prado-Novoa et al., 2019), which might not have the same suite of adjustments that are possible in the field. For example, although walking over variable terrain increases metabolic costs overall (Voloshina et al., 2013), increased stability has the ability to modify the difficulty, making dynamic surfaces easier to negotiate (Hawkins et al., 2017). Our expectation is that the fine-tuning of kinematics that can occur on a treadmill is exactly what allows humans to maintain such metabolically effective locomotion (Wall-Scheffler & Myers, 2013). Constraining the speed walked is probably why only equal efficiency was seen in prior studies, rather than increased efficiency for women when able to modulate their gait and speed. It has been elegantly shown that ‘imposed constraints disrupt the normal gait pattern by interfering with the natural control system’ and that constraints can be in the form of speed, as well as speed–frequency relationships with step patternings typical of what happens on a treadmill (Bertram, 2005). Managing all of our optimisation studies on treadmills might be preventing us from elucidating key mechanisms relating to altered stability (Gast et al., 2019; Voloshina et al., 2013), the flexibility of footfall patterns (Bertram, 2005; Lee et al., 2013; Ruina et al., 2005) and changes in soft tissue work (Zelik & Kuo, 2010) that allow the long distance mobility that humans have evolved to accomplish. Indeed, studies of speed changes on and around treadmills find that the mechanical work alone does not explain the metabolic cost perturbations (Minetti et al., 2001), and there are probably soft tissue, as well as kinematic, adjustments that must happen to create the energy saving mechanisms that people employ when modulating their speed to manage variable terrain (Gast et al., 2019). Thus in summary, although the overall costs of walking overground might be higher, the ability to fine-tune and optimise to one's specific morphology might be highlighted when walking overground – thus finally elucidating why humans may have evolved the specific body size, shape and composition that sets us apart from other apes (Reno, 2014). Many more studies need to be done off the treadmill for us to fully appreciate the range of mobility options people have (e.g. Rue & Kramer, 2017), and the specific selection pressures acting on human morphology.

At this point, we can be confident that people evolved to walk overground while carrying loads (Bennett et al., 2009, 2020; Brooks et al., 2018; Potts, 1988), the two selection pressures specifically measured here. Some additional comments about the loaded positions not covered in this experimental design, namely tumpline carrying and head loads, can be mentioned here. Both of these loaded positions have been shown to allow people practised in these styles to carry remarkably heavy loads for virtually no increase in cost (Maloiy et al., 1986; Minetti et al., 2006). Interestingly, investigations into how people are able to carry habitual loads effectively generally involve discussions of training, fitness, etc., but also always end with the assessment that increased stabilisation and the ability to maintain balance while loaded with reduced muscular work are probably what lead to increased efficiency and economy (Minetti et al., 2006). Thus, although these studies may not specifically look at measures of pelvis width or sex differences, and although they are looking at different loading positions than here, the basic physics of carrying loads well do distil to the same issues – being able to maintain stability and utilise a practised central pattern generator to exchange energy through the gait cycle will result in lower metabolic costs.

Given these findings, it is clear that people are excellent load carriers! For most typical loading positions (those tested here), the increased cost is similar regardless of whether the mass is symmetrical (i.e. front or back) or asymmetrical (i.e. on the side). In fact, a 7.6% incline is more challenging than a 16% load! The ways in which humans navigate the intersection between these variables in field situations should be the focus of future work.

## Research transparency

All data are available from CMWS upon request.
